# Minimally Invasive Unilateral Lumbar Decompression in the Lateral Position Under Spinal Anesthesia: A Retrospective Study

**DOI:** 10.7759/cureus.92392

**Published:** 2025-09-15

**Authors:** Maya Ghazi, Melissa Matar, Jad Al Masri, Elie Fahed, Ali Msheik, Philippe Younes

**Affiliations:** 1 Neurology, Lebanese American University Medical Center, Beirut, LBN; 2 Faculty of Medical Sciences, Lebanese University, Beirut, LBN; 3 Neurological Surgery, Bellevue Medical Center, Beirut, LBN; 4 Neurosurgery, Hamad Medical Corporation, Doha, QAT

**Keywords:** lebanon, lumbar spinal stenosis, minimally invasive, spinal anesthesia, spine surgery

## Abstract

Introduction

Lumbar spinal stenosis (LSS) is a debilitating condition characterized by the narrowing of the lumbar spinal canal, often affecting older adults. This study evaluates the efficacy and clinical outcomes of a novel minimally invasive decompression technique via a unilateral approach under spinal anesthesia in a lateral position.

Methods

A retrospective monocentric observational study was conducted from January 2023 to January 2024, including 74 patients who underwent surgery between 2018 and 2023. Data was collected through a 30-question survey, utilizing validated scales from the literature, and analyzed using the IBM SPSS Statistics for Windows, Version 25 (Released 2017; IBM Corp., Armonk, New York, United States).

Results

The average patient age was 69, with 47 males and 27 females. Most patients were uneducated (n = 39), and half experienced maximal pain before surgery (n = 33). One month post-surgery, 30 patients had no pain, and 24 had minimal pain. Education in non-healthcare fields was associated with greater improvement in the Oswestry Disability Index (ODI) (p = 0.037). Four patients had a dural tear. The average surgery duration was 105 minutes, ranging from 70 to 190 minutes.

Conclusion

This minimally invasive technique offers significant pain relief and improved disability outcomes, suggesting a quicker recovery and better quality of life for patients. Further research is needed to refine patient selection, optimize surgical techniques, and evaluate long-term outcomes.

## Introduction

Lumbar spinal stenosis (LSS) is a prevalent degenerative condition primarily affecting the elderly, characterized by a narrowing of the spinal canal that compresses neural elements and manifests as neurogenic claudication, numbness, and lower limb weakness [1,2]. The etiology involves progressive degeneration, ligamentum flavum thickening, and facet joint hypertrophy, often exacerbated by sedentary lifestyles and age-related spinal wear [3,4].

While initial management includes physical therapy, nonsteroidal anti-inflammatory drugs (NSAIDs), and epidural steroid injections, surgical decompression remains the definitive treatment when conservative approaches fail [5,6]. Traditional laminectomy, although effective in decompressing the neural elements, carries the risk of significant tissue trauma, paraspinal muscle disruption, prolonged recovery, and potential need for spinal fusion [7,8].

In response, minimally invasive surgery (MIS) has emerged as a refined approach, aiming to reduce iatrogenic damage while maintaining decompression efficacy. MIS techniques, particularly unilateral laminotomy for bilateral decompression (ULBD), preserve posterior spinal elements and reduce blood loss, infection rates, and hospital stays [9,10]. Technological advances in imaging, instrumentation, and intraoperative neuromonitoring have further facilitated the adoption of endoscopic spine surgery [11].

Notably, a technical variant, unilateral MIS for bilateral decompression under spinal anesthesia in the lateral position, has gained traction for its reduced morbidity and faster functional recovery [11]. Spinal anesthesia offers advantages over general anesthesia, including lower pulmonary complication rates and enhanced perioperative hemodynamic stability.

Despite its clinical potential, this surgical approach remains underutilized in Lebanon, and published outcomes are limited. This study seeks to evaluate the efficacy and perioperative profile of this MIS variation as performed at Bellevue Medical Center.

## Materials and methods

Study design and population

This study is a retrospective observational study including 74 patients. Data were extracted from patients’ records at the Bellevue Medical Center (BMC) from 2018 to 2023. The study was conducted between January 2023 and January 2024. From the patients registered in the data at BMC, all patients who had undergone minimally invasive bilateral decompression via unilateral approach under spinal anesthesia and lateral approach, and met the inclusion criteria, were enrolled in this study.

Inclusion Criteria

All patients who had minimally invasive bilateral decompression of lumbar stenosis via unilateral approach under spinal anesthesia in a lateral recumbent position were included. The subjects included in the study were from different Lebanese regions.

Exclusion Criteria

Key exclusion criteria included patients who underwent discectomy in addition to decompression, prior surgery, open or conventional surgery, general anesthesia, or fusion. The patients who had missing information in their medical files were also excluded, resulting in the exclusion of 48 patients out of a total of 127 screened. Eleven patients were excluded due to a history of prior surgery, and four patients required general anesthesia.

Steps of the surgery

**Figure 1:**
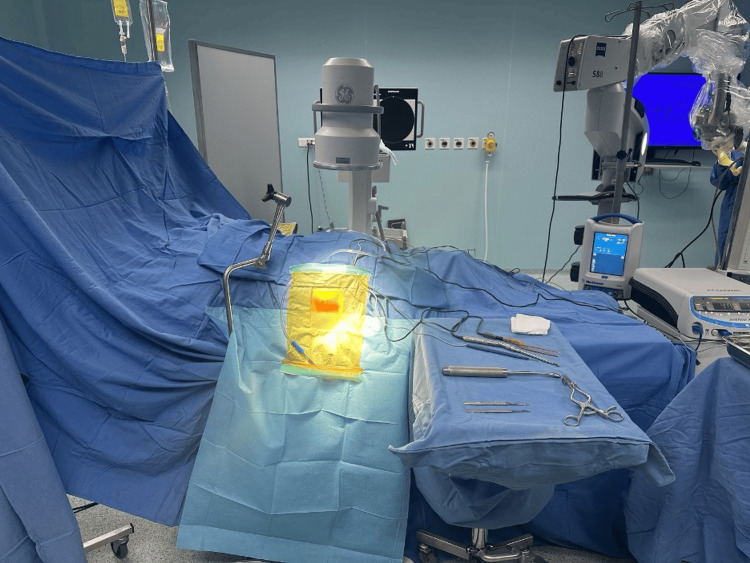
Intraoperative positioning of the patient after scrubbing and draping.

**Figure 2:**
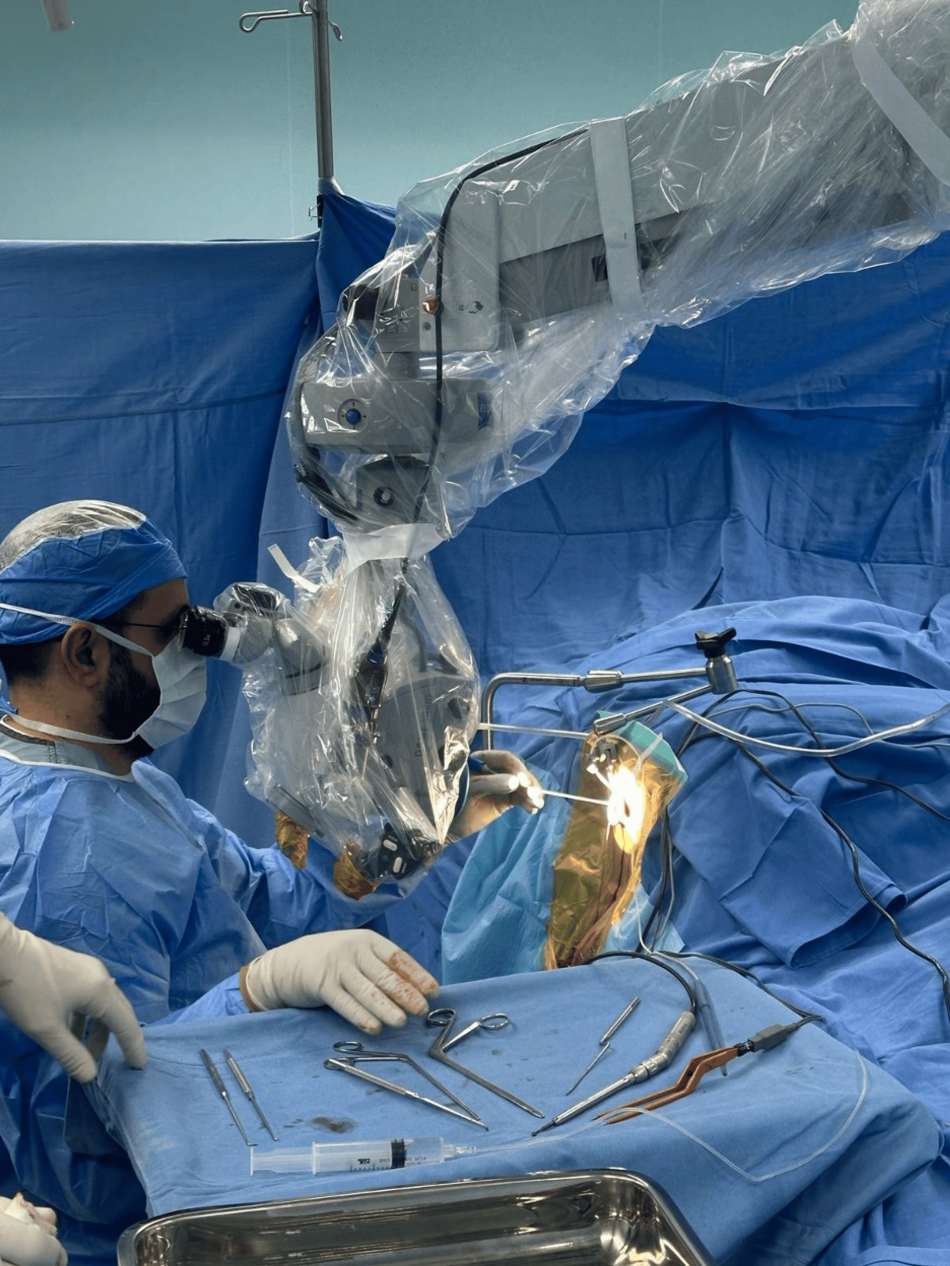
The position of the surgeon during the procedure.

**Figure 3:**
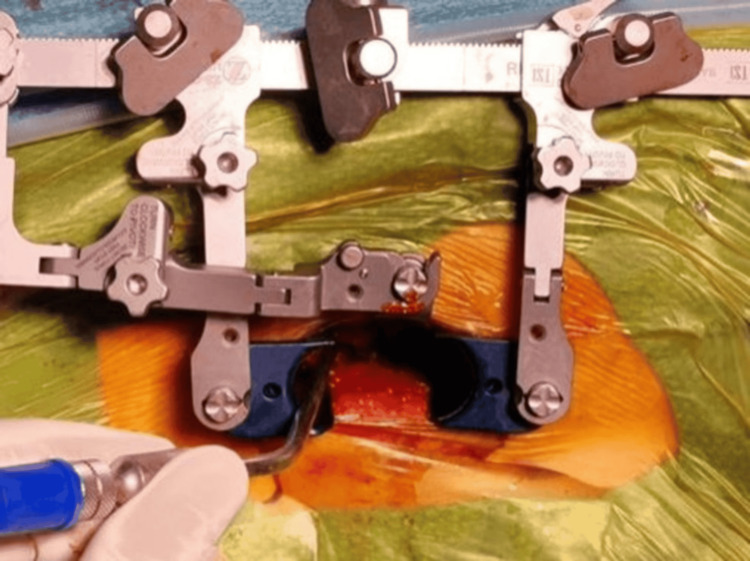
The dilator and fixation system used for the field procedure.

**Figure 4:**
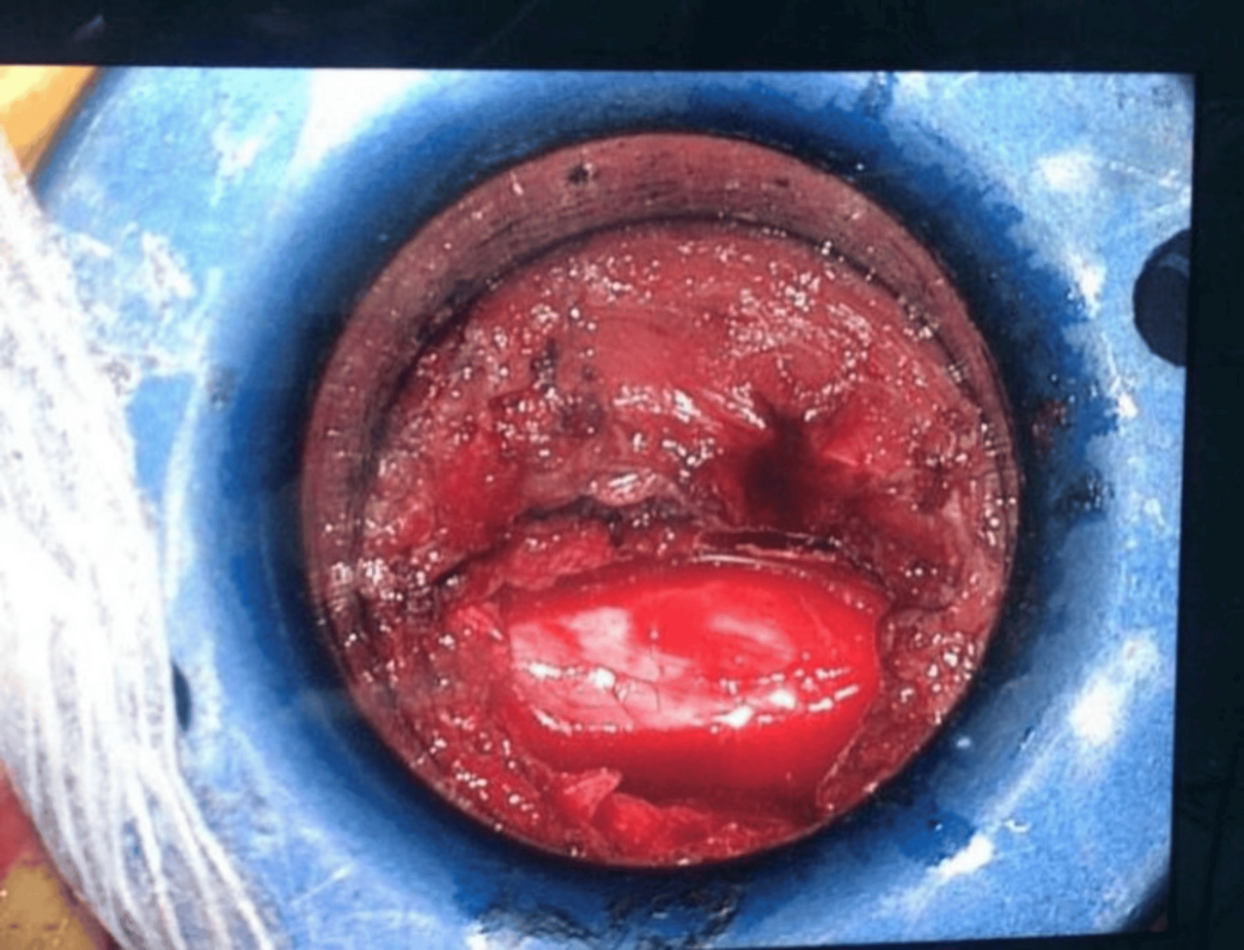
Appearance of the site of surgery through the field.

**Figure 5:**
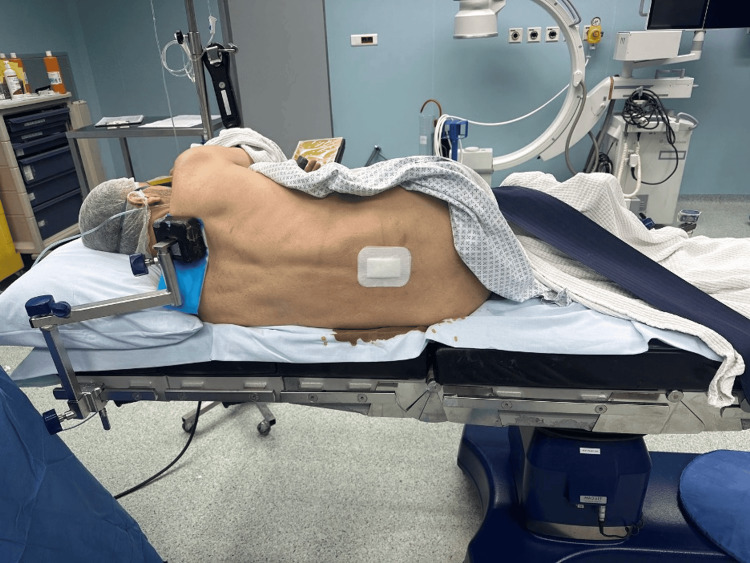
The positioning of the patient with the dressing applied. Of note, this is the position of the patient before and during the surgery. The knees are cushioned and flexed.

Steps of the surgical technique followed at BMC are detailed in Appendix 1 and depicted in Figures 1-5. 

Sample size

The sample size was determined using G*Power software (Heinrich-Heine-Universität Düsseldorf, Düsseldorf, Germany). The calculation was performed for a two-tailed test with an effect size of 0.9153404, an alpha error probability (α) of 0.001, and a power (1-β) of 0.80. The output parameters included a no centrality parameter of 4.6673386, a critical t-value of 1.9599640, and 25 degrees of freedom. As a result, the total sample size required for the study was estimated to be 26, yielding an actual power of 0.8072813 (Appendix 2).

Data collection 

Data was collected from the archive of BMC. A survey developed and adopted from validated scales from the literature was used to collect data. The questionnaire was a set of 30 questions (Appendix 3). The sections included details about the patients’ demographics, preoperative symptoms, surgical details, and the postoperative follow-up for claudication and the related pain using the visual analogue score (VAS). The questions provided the total data needed to evaluate the efficiency and efficacy of this type of surgery. This questionnaire helped to analyze the outcome of such a procedure and to highlight its success by comparing it to conventional surgical procedures in the literature.

Data analysis

Statistical analysis was done using the IBM SPSS Statistics for Windows, Version 25 (Released 2017; IBM Corp., Armonk, New York, United States). A descriptive analysis was made, where qualitative data (nominal) were represented as frequencies and percentages, and quantitative data (scale) were represented by mean, standard deviation, median, minimum, and maximum.

Bivariate analysis was conducted, aiming to test the factors associated with the knowledge score. The tests used in the bivariate analysis were the independent t-test, ANOVA test, and Pearson correlation test. A statistically significant association was set at 5% (p-value less than 0.05). A multiple linear regression was used to test the factors associated with the knowledge score. The adjusted multiple linear regression model was conducted via the stepwise model.

A bivariate analysis was carried out to identify the correlation between claudication/disability and sociodemographic factors and to compare claudication and disability rates pre- and post-op. The students’ test was utilized to compare means between two groups, and the ANOVA test was used to compare means between more than two groups. A paired sample t-test was used to compare means at two different durations. A p<0.05 was considered statistically significant.

A multinomial logistic regression was conducted, taking the levels of claudication/disability as the dependent variable. All variables with a p<0.2 were included in the final model as independent variables, including age (in years) and gender for the claudication model and age, education, residency, and occupation for the disability model. The significance level was set at p<0.05.

Approval for usage of the Oswestry Disability Index (ODI) was granted through "https://eprovide.mapi-trust.org/instruments/oswestry-disability-inde" under the number 119615. 

Ethical considerations

This retrospective study was conducted concerning the ethical principles of the Declaration of Helsinki, which was developed originally in 1964. Institutional Review Board (IRB) approval was granted by the ethical committee of the BMC.

Confidentiality and anonymity of all data and participants were assured, and patients’ names were replaced by study codes.

## Results

Demographics

The average age of patients was around 69 years, and the majority were males, making up around two-thirds of the sample (63.5%; n = 47). Around 86.5% (n = 64) were married, and 77% (n = 57) were residing in Beirut. More than half of the patients were not educated (52.7%; n = 39), while only 13.5% (n = 10) had a healthcare-related education (Table 1).

**Table 1:** Sociodemographic characteristics of included patients.

Factor	Category	Number	Percentage (%)
Gender	Female	27	36.5
	Male	47	63.5
Marital Status	Married	64	86.5
	Single	8	10.8
	Divorced	1	1.4
	Widowed	1	1.4
Residency Location	Beirut	57	77
	North	8	10.8
	South	2	2.7
	Bekaa	6	8.1
	Mount Lebanon	1	1.4
Education	Non-healthcare education level	25	33.8
	Healthcare-related education	10	13.5
	Not educated	39	52.7

Occupation

Around half of the patients were unemployed (48.6%; n = 36), a slightly smaller proportion had an occupation requiring little or no physical effort (40.5%), while only 10.8% (n = 8) had an occupation requiring physical effort.

Quality of life before surgery 

All patients had pain, where a third had moderate pain (n = 22), around half had severe pain (n = 36), and the rest had very severe or the worst imaginable pain (n = 16) (Table 2). As for disability levels, none were able to look after themselves, lift heavy things, walk, sit, stand, have a social life, and travel without pain (n = 0), while only five patients were able to sleep without being disturbed by pain. Around a third needed help in all aspects of life (n = 21), and around 40% could not carry or lift anything (n = 29) and were restricted to short journeys and travels under 30 minutes (n = 30). Due to pain, half of the patients were not able to walk more than 500 meters (n = 37), sit for more than an hour (n = 38), stand without pain (n = 34), or go out often (n = 37). One-fifth of patients slept less than four hours (n = 16), and another one-fifth slept less than two hours (n = 15) due to pain.

**Table 2:** Assessment of the QoL before and after surgery using the Oswestry Disability Index (ODI) scale. SD: standard deviation; QoL: quality of life

Category	Characteristics	Before Surgery			After Surgery		
		Number	Percentage	Mean ± SD	Number	Percentage	Mean ± SD
Pain Intensity	I had no pain	0	0	3.014 ± 0.899	59	79.7	0.203 ± 0.405
	Pain was very mild	0	0		15	20.3	
	Pain was moderate	22	29.7		0	0	
	Pain was fairly severe	36	48.6		0	0	
	Pan was very severe	9	12.2		0	0	
	Worst imaginable pain	7	9.5		0	0	
Personal Care	Can look after myself normally without extra pain	0	0	2.392 ± 1.353	64	86.5	0.284 ± 0.803
	Can look after myself normally, but with extra pain	25	33.8		4	5.4	
	Painful to look after myself, I am slow and careful	17	23		1	1.4	
	Need some help, but manage most of my personal care	0	0		5	6.8	
	Need help every day in most aspects of self-care	21	28.4		0	0	
	Do not get dressed, I wash with difficulty and stay in bed	11	14.9		0	0	
Lifting	Can lift heavy weights without extra pain	0	0	3.014 ± 1.802	38	51.4	1.514 ± 1.746
	Can lift heavy weights with extra pain	29	39.2		3	4.1	
	Pain prevents me from lifting heavy weights off the floor, but can manage if they are conveniently placed	2	2.7		11	14.9	
	Pain prevents me from lifting heavy weights, but I can manage light to medium weights if they are conveniently positioned	11	14.9		1	1.4	
	I can lift very light weights	3	4.1		21	28.4	
	I cannot lift or carry anything at all	29	39.2		0	0	
Walking	Pain does not prevent me from walking any distance	0	0	2.703 ± 1.132	59	79.7	0.324 ± 0.742
	Pain prevents me from walking more than 2 kilometers	15	20.3		8	10.8	
	Pain prevents me from walking more than 1 kilometre	10	13.5		0	0	
	Pain prevents me from walking more than 500 metres	37	50		6	8.1	
	I can only walk using a stick or crutches	6	8.1		1	1.4	
Sitting	I am in bed most of the time	6	8.1	2.568 ± 1.124	0	0	0.149 ± 0.358
	I can sit in any chair as long as I like	0	0		63	85.1	
	I can only sit in my favourite chair as long as I like	9	12.2		11	14.9	
	Pain prevents me from sitting for more than one hour	38	51.4		0	0	
	Pain prevents me from sitting for more than 30 minutes	7	9.5		0	0	
	Pain prevents me from sitting for more than 10 minutes	16	21.6		0	0	
	Pain prevents me from sitting at all	4	5.4		0	0	
Standing	I can stand as long as I want without extra pain	0	0	2.311 ± 1.471	60	81.1	0.284 ± 0.631
	I can stand as long as I want, but it gives me extra pain	34	45.9		7	9.5	
	Pain prevents me from standing for more than 1 hour	9	12.2		7	9.5	
	Pain prevents me from standing for more than 3 minutes	16	21.6		0	0	
	Pain prevents me from standing for more than 10 minutes	4	5.4		0	0	
	Pain prevents me from standing at all	11	14.9		0	0	
Sleeping	My sleep is never disturbed by pain	5	6.8	3.230 ± 1.522	50	67.6	0.351 ± 0.535
	My sleep is occasionally disturbed by pain	5	6.8		22	29.7	
	Because of pain, I have less than 6 hours of sleep	13	17.6		2	2.7	
	Because of pain, I have less than 4 hours of sleep	16	21.6		0	0	
	Because of pain, I have less than 2 hours of sleep	15	20.3		0	0	
	Pain prevents me from sleeping at all	20	27		0	0	
Social Life	My social life is normal and gives me no extra pain	0	0	2.946 ± 1.097	60	81.1	0.216 ± 0.504
	My social life is normal, but it increases the degree of pain	10	13.5		13	17.6	
	Pain has no significant effect on my social life apart from limiting my more energetic interests, e.g: sport	9	12.2		1	1.4	
	Pain has restricted my social life, and I do not go out as often	37	50		0	0	
	Pain has restricted my social life to my home	11	14.9		0	0	
	I have no social life because of pain	7	9.5		0	0	
Travelling	I can travel anywhere without pain	0	0	3.351 ± 1.176	59	79.7	0.203 ± 0.405
	I can travel anywhere, but it gives me extra pain	11	14.9		15	20.3	
	Pain is bad, but I manage journeys over two hours	0	0		0	0	
	Pain restricts me to journeys of less than one hour	24	32.4		0	0	
	Pain restricts me to short necessary journeys under 30 minutes	30	40.5		0	0	
	Pain prevents me from travelling except to receive treatment	9	12.2		0	0	
Total ODI score		56.727 ± 13.390			7.838 ± 7.276		

Quality of life after surgery 

The pain was assessed according to the VAS subjective inference of each patient. Consistent follow-up data were available at two weeks, where around 80% of patients had no pain (n = 59), and the remaining had very mild pain (n = 15). Longer-term pain data were inconsistently reported across studies and thus not amenable to pooled analysis. In contrast, claudication and disability outcomes were more consistently reported at medium-term follow-up (three to six months) and are presented accordingly. As for disability levels, 86.5% were able to look after themselves (n = 64), 51.4% were able to lift heavy things (n = 38), 79.7% were able to walk any distance (n = 59), 85.1% were able to sit for any period (n = 63), 81.1% were able to stand for any period (n = 60), 67.6% were not disturbed in their sleep (n = 50), 81.1% had a normal social life (n = 60), and 79.7% could travel without pain (n = 59) (Table 2).

ODI score: change following surgery

Pain intensity, lifting, walking, standing, and sleeping abilities were all significantly improved following surgery at the defined follow-up interval (two weeks for pain, three to six months for functional outcomes). Pain intensity decreased from 3.014 ± 0.899 to 0.203 ± 0.405 (mean difference: 2.811; p = 0.028), lifting from 3.014 ± 1.802 to 1.514 ± 1.746 (mean difference: 1.500; p<0.001), walking from 2.703 ± 1.132 to 0.324 ± 0.742 (mean difference: 2.379; p<0.001), standing from 2.311 ± 1.471 to 0.284 ± 0.631 (mean difference: 2.027; p = 0.002), and sleeping from 3.230 ± 1.522 to 0.351 ± 0.535 (mean difference: 2.879; p = 0.009). As for the overall ODI score, assessed at three to six months, it improved markedly, with a reduction from 56.727 ± 13.390 preoperatively to 7.838 ± 7.276 postoperatively (p = 0.000) (Table 3).

**Table 3:** The change in Oswestry Disability Index following surgery. ODI: Oswestry Disability Index [12]. Approval for usage of the ODI was granted through "https://eprovide.mapi-trust.org/instruments/oswestry-disability-inde" under the number 119615. * denotes statistically significant p-values

Category	Pre op	Post op	P-value
	Mean ± SD	Mean ± SD	
Pain Intensity	3.014 ± 0.899	0.203 ± 0.405	0.028*
Personal Care	2.392 ± 1.353	0.284 ± 0.803	0.469
Lifting	3.014 ± 1.802	1.514 ± 1.746	0.000*
Walking	2.703 ± 1.132	0.324 ± 0.742	0.000*
Sitting	2.568 ± 1.124	0.149 ± 0.358	0.426
Standing	2.311 ± 1.471	0.284 ± 0.631	0.002*
Sleeping	3.230 ± 1.522	0.351 ± 0.535	0.009*
Social Life	2.946 ± 1.097	0.216 ± 0.504	0.856
Travelling	3.351 ± 1.176	0.203 ± 0.405	0.362
ODI Score	56.727 ± 13.390	7.838 ± 7.276	0.000*

Disability levels

**Figure 6:**
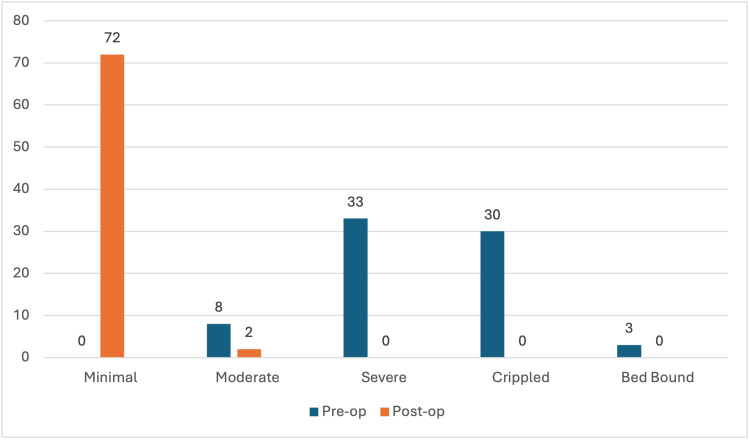
Comparison of the disability level before surgery and two weeks after surgery.

No patients had a minimal disability before surgery, while around 45% had severe disability levels (n = 33) and 41% had a crippled disability (n = 30). Only eight patients had moderate disability, and three were bed-bound. None had minimal disability. However, all patients had minimal disability two weeks following the surgery, except for two who had moderate disability (Figure 6).

Claudication pain assessment before surgery

**Figure 7:**
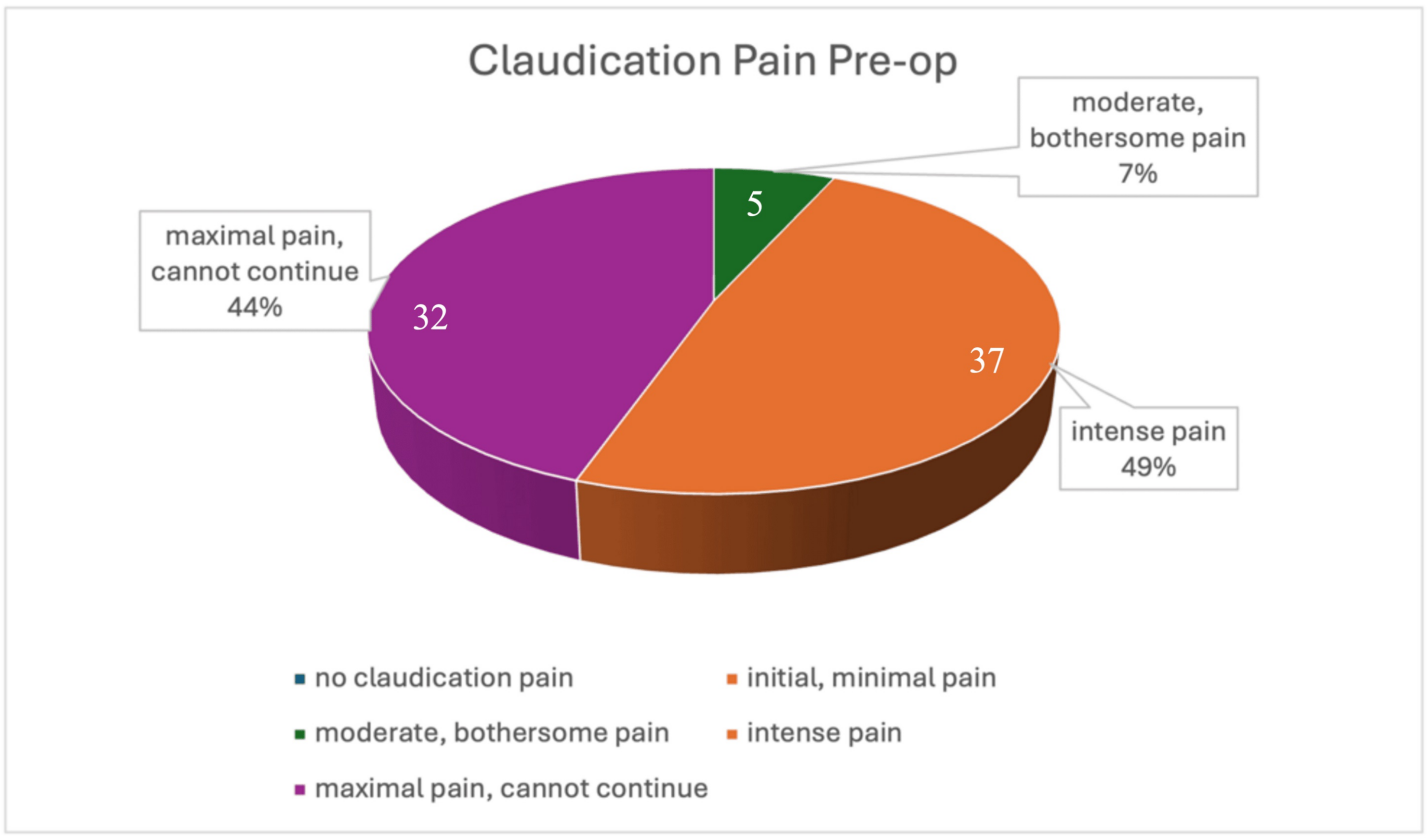
Claudication assessment before the surgery.

Around half of the patients had intense pain, 44% had maximal pain, and the remaining 7% had moderate pain. None had minimal or no pain (Figure 7).

Claudication pain assessment post-op

**Figure 8:**
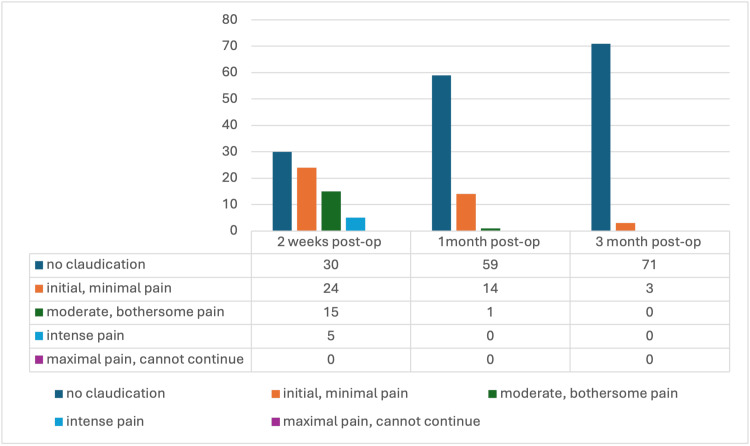
Evolution of claudication at two weeks, one month, and three months after the surgery.

Figure 8 shows the claudication pain levels two weeks, one month, and three months following the surgery. Two weeks post-surgery, 30 patients had no claudication pain, 24 had mild pain, 15 had moderate pain, five had intense pain, and none had maximal pain. These rates decreased one month and six months post-surgery, where the majority had minimal pain (59 and 71 patients, respectively). None had intense or maximal pain one month or six months after the surgery.

Surgical characteristics and complications

Around a third of patients had surgery at the level of L4-L5 and another third at the level of L3-L5 (Table 4). Around a third had surgery at one level of the vertebrae, 40.5% at two levels of the vertebrae, and the remaining 8.1% at three levels of the vertebrae. No patients had significant intraoperative bleeding, with the mean estimated blood loss recorded as 5 mL (Table 4). No patient developed postoperative infection. Only four patients experienced dural tears (5.4%). These were managed intraoperatively through the minimally invasive approach, with primary suture repair performed when feasible, and fibrin glue with fat/fascial graft packing applied in cases where direct suturing could not be achieved. The average duration of surgery was approximately 105 minutes, ranging from 70 to 190 minutes.

**Table 4:** Description of surgical characteristics and complications. SD: standard deviation

Factor	Category	Number	Percentage (%)
Vertebral Level	L3-L4	2	2.7
	L4-L5	26	35.1
	L5-S1	10	13.5
	L2-L4	4	5.4
	L3-L5	22	29.7
	L4-S1	4	5.4
	L2-L5	6	8.1
Number of Levels	One level	38	51.4
	Two levels	30	40.5
	Three levels	6	8.1
Bleeding	Yes	0	0
	No	74	100
Dural Tear	Yes	4	5.4
	No	70	94.6
Post-op Infection	Yes	0	0
	No	74	100
Operation Time (min)	Mean ± SD	104.5 ± 17.8	
	Min-Max	70	190

Bivariate analysis

Sociodemographic Factors

Among the sociodemographic factors analyzed, education level was the only variable significantly associated with ODI improvement. Patients without healthcare-related education showed the greatest functional improvement (mean ODI difference = 53.60 ± 8.27), while those with healthcare-related backgrounds exhibited the least improvement (mean ODI difference = 42.44 ± 9.74), with the difference reaching statistical significance (p = 0.037). In contrast, claudication improvements did not differ significantly across education groups (p = 0.556).

Other demographic factors such as age (ODI: R = 0.143, R² = 0.02, p = 0.226), gender (male vs female: 48.94 ± 10.48 vs 48.81 ± 11.24, p = 0.960), marital status (p = 0.754), residency location (p = 0.204), and occupation type (p = 0.266) were not significantly associated with changes in ODI scores. Similarly, no statistically significant differences were observed in claudication improvement across any of these variables, including gender (p = 0.190), marital status (p = 0.621), residency (p = 0.871), or occupation (p = 0.779), as detailed in Table 5.

**Table 5:** The effect of socio-demographics on the improvement in claudication pain and ODI scores following surgery. ODI: Oswestry Disability Index; SD: standard deviation * denotes statistically significant p-values

Factor	Category	Claudication Difference		ODI Difference	
		Mean ± SD	P-value	Mean ± SD	P-value
Age	R (R-squared)	0.173 (0.03)	0.141	0.143 (0.02)	0.226
Gender	Female	2.259 ± 0.813	0.190	48.807 ± 11.238	0.960
	Male	2.553 ± 0.974		48.936 ± 10.480	
Marital Status	Married	2.484 ± 0.926	0.621	48.819 ± 10.543	0.754
	Single	2.375 ± 0.916		53.056 ± 9.682	
	Divorced	1.000		26.667	
	Widowed	2.000		42.222	
Residency Location	Beirut	2.474 ± 0.947	0.871	48.460 ± 10.653	0.204
	North	2.250 ± 0.707		53.611 ± 11.665	
	South	3.000		37.778 ± 15.713	
	Bekaa	2.167 ± 1.169		49.260 ± 7.751	
	Mount Leban	3.000		55.556	
Education	Non-healthcare education level	2.320 ± 0.802	0.556	53.600 ± 8.273	0.037*
	Healthcare-related education	2.600 ± 1.075		42.444 ± 9.740	
	Not educated	2.487 ± 0.970		47.521 ± 11.235	
Occupation	Unemployed	2.417 ± 0.937	0.779	49.877 ± 11.337	0.266
	Occupation requiring little or no physical effort	2.433 ± 0.935		47.333 ± 10.311	
	Occupation requiring physical effort	2.625 ± 0.916		9.498	

Vertebral Levels

Table 6 summarizes the effect of the specific vertebral level involved in the surgery and the number of treated vertebrae on improvements in claudication pain and disability. However, no significant associations were found. For claudication improvement, mean differences ranged from 1.50 ± 0.71 (L3-L4) to 3.00 ± 0.82 (L4-S1), with p = 0.449. For ODI improvement, mean differences ranged from 45.93 ± 6.69 (L2-L5) to 58.89 ± 4.71 (L3-L4), with p = 0.747, indicating no statistically meaningful variation by vertebral level. Similarly, the number of treated vertebrae (1, 2, or 3 levels) did not significantly affect postoperative outcomes. Claudication improvement scores were comparable across groups (p = 0.727), and ODI scores also showed no significant differences (p = 0.680). These findings suggest that neither the number nor the specific levels of operated vertebrae significantly influenced postoperative functional or pain outcomes (Table 6).

**Table 6:** The effect of vertebral levels on the improvement in claudication pain and ODI scores following surgery. ODI: Oswestry Disability Index; SD: standard deviation

Factor	Category	Claudication Difference		ODI Difference	
		Mean ± SD	P-value	Mean ± SD	P-value
Vertebral Level	L3-L4	1.50 ± 0.707	0.449	58.888 ± 4.714	0.747
	L4-L5	2.423 ± 0.986		48.205 ± 12.053	
	L5-S1	2.70 ± 0.823		52.0 ± 8.523	
	L2-L4	2.0 ± 0.816		50.555 ± 11.949	
	L3-L5	2.50 ± 0.963		47.676 ± 10.966	
	L4-S1	3.0 ± 0.816		50.0 ± 12.103	
	L2-L5	2.167 ± 0.752		45.925 ± 6.691	
Number of Treated Vertebrae	1	2.447 ± 0.950	0.727	49.766 ± 11.135	0.680
	2	2.5 ± 0.937		48.37 ± 10.881	
	3	2.167 ± 0.752		45.925 ± 6.691	

Multivariate (mv) analysis

MV Analysis ODI Difference

The multinomial regression analysis examining the change in disability index from preoperative to two weeks postoperative (Table 7) identified education as the only significant predictor. Specifically, patients with non-healthcare-related education demonstrated significantly higher disability scores postoperatively compared to others (B = 8.464, p = 0.008, 95% CI: 2.248-14.680). In contrast, healthcare-related education (B = -2.262, p = 0.611), age (B = 0.440, t = 0.367), residency location (B = 0.449, t = 0.366), and occupation (B = -2.860, p = 0.197) were not significantly associated with disability improvement.

**Table 7:** Multiple linear regression taking the difference in disability index. CI: confidence interval

Category	Unstandardized Coefficients		Standardized Coefficients	t	Sig.	95% CI for B	
	B	Std. Error	Beta			Lower Bound	Upper Bound
Constant	44.860	9.031		4.967	0.00	26.839	62.882
Age	0.440	0.121	0.049	0.367	0.715	-0.197	0.285
Residency Location	0.449	1.225	0.041	0.366	0.715	-1.996	2.893
Education (non-healthcare related vs. others)	8.464	3.115	0.377	2.717	0.008	2.248	14.680
Education (healthcare-related vs. others)	-2.262	4.429	-0.073	-0.511	0.611	-11.100	6.576
Occupation	-2.860	2.195	-0.181	-1.303	0.197	-7.240	1.521

MV Analysis for the Claudication Difference

Table 8 presents the results of a multinomial regression analysis evaluating the change in claudication levels from before surgery to two weeks after the procedure. Neither gender nor age demonstrated a statistically significant association with claudication improvement. Specifically, male gender (vs. female) had no significant effect (B = 0.267, p = 0.232, 95% CI: -0.175 to 0.708), and age similarly showed no significant predictive value (B = -0.013, p = 0.172, 95% CI: -0.031 to 0.006).

**Table 8:** Multiple linear regression taking the difference in claudication pain. Dependent variable: Difference in claudication levels between pre-surgery and two weeks post-surgery

Category	Unstandardized Coefficients		Standardized Coefficients	t	Sig.	95% CI	
	B	Std. Error	Beta			Lower Bound	Upper Bound
Constant	3.144	0.664		4.731	0.000	1.819	4.469
Gender Male vs. Female	0.267	0.221	0.140	1.205	0.232	-0.175	0.708
Age	-0.013	0.009	-0.160	-1.381	0.172	-0.031	0.006

## Discussion

LSS is considered one of the most common indications for spinal surgery, representing a leading cause of operative intervention in the aging population. Several surgical techniques are available, with possible differences in results and complications. By assessing the benefits of minimally invasive bilateral decompression of lumbar stenosis by unilateral approach, this study showed that disability levels were significantly improved following surgery in most daily actions, and pain levels were significantly decreased. No cases were complicated by bleeding or bacterial infections, while 5.4% of cases had dural tears during surgery [12].

In this sample, the average age of patients was 68 years, with 63.5% being males. The incidence of LSS differs in each age group. Multiple population-based studies confirmed that the incidence of LSS increases with age, 1.7-2.2% in the population aged between 40-49 years of age and 10.3-11.2% in those between 70-79 years of age [13].

Even though females develop a higher level of disability than males [12,14], different studies proved that it does not differ significantly, where surgical decompression, being open or minimally invasive, is safe and effective. Numerous past studies correlate an optimal operative time, a reasonable amount of bleeding, and reduced iatrogenic soft tissue damage, and good long-term outcomes (including postoperative pain) to minimally invasive bilateral decompression [15]. However, these two approaches have similar complication profiles [15]. Conducting their cohort study, Rahman et al. estimated the complication rate of minimally invasive lumbar laminectomy for lumbar stenosis of approximately 7.9%, compared to 16.1% in the open technique group [16]. Additionally, the most encountered complication is postoperative infection (or radiculitis), having an incidence ranging between 2.8 and 57.1% [17]. Even so, the patients enrolled in this study didn’t develop any postoperative infection. As for the incidental durotomy, it is the third most common complication, with an incidence range of 0.3-8.6% [17]. This is comparable to the results of this study, where 5.4% had a dural tear during the surgery.

It has been asserted that, regardless of the surgical technique type, the extent of muscle injury is highly influenced by the average duration of surgery [18], 105 minutes in this study compared to 90 minutes in similar studies [18]. Compared to open surgeries, this duration is longer, whereas the comparative study found that MIS was 11 minutes longer on average [15]. As for blood loss, the mean amount in minimally invasive bilateral decompression was approximately equal to 40 ml in this study, which is considerably less than that reported in open surgeries (around 110 ml) [16]. 

It’s quite confusing for the postoperative patient to measure the outcomes of spine surgery [19]. Even though minimally invasive bilateral decompression leads to an important improvement mainly in the ODI score [17,20], the literature lacks enough studies that compare its results to those of a conventionally treated group by an open surgery. Additionally, a 10-point improvement in ODI score is a clinically significant improvement. Mobbs et al. showed that, regardless of the important improvements in function (using the ODI score) ensured by the minimally invasive and open lumbar surgery, neither approach was proven to be superior to the other [10]. In this study, the pain intensity, lifting, walking, standing, and sleeping abilities were all significantly improved following surgery. Furthermore, a similar study showed that, during the immediate postoperative period, the minimally invasive group of patients had better mobility than the conventional open group [21].

It was demonstrated by several previous studies that a significant improvement in the functional outcome as well as pain scores is achieved by a single-level MIS [15,22]. However, it has been demonstrated that multilevel surgical techniques are clinically successful and have low rates of complications [23,24]. This study confirmed the effectiveness of minimally invasive decompression in reducing claudication within the first month, assuring its rapid effect.

This study expands the range of treatment options available by demonstrating that minimally invasive bilateral decompression of lumbar stenosis via a unilateral approach under spinal anesthesia is associated with favorable outcomes in terms of pain relief and reduced disability. While these results highlight the potential benefits of this approach, no direct comparison with conventional techniques was made; therefore, no conclusions regarding relative superiority can be drawn. Future comparative studies are warranted to further validate these findings.

In addition, this technique appears to reduce healthcare burden by shortening hospital stays and lowering postoperative care requirements, potentially leading to cost savings for healthcare systems and easing pressure on hospitals and rehabilitation facilities. The favorable outcomes are also associated with high patient satisfaction, driven by rapid symptom improvement and early return to normal activities. Finally, this study opens avenues for further research into refining patient selection, optimizing surgical techniques, and evaluating long-term outcomes. Given the scarcity of research in this field in developing countries in general and in Lebanon specifically, our findings provide added value by assessing the role of minimally invasive techniques within specific sociodemographic contexts.

Limitations

Despite having several points of strength, this study holds some limitations. For instance, the retrospective design of this study, with a small sample from a single hospital, makes it hard to generalize the results reached. In addition, the absence of a direct comparison with other decompression techniques, particularly conventional surgery, limits the strength of our conclusions regarding the relative advantages of this minimally invasive approach. Future comparative studies are required to better delineate these potential benefits. Furthermore, this study could have some confounding variables that may affect the results, such as the surgeon’s experience. For instance, the assessment of past medical history and the presence of certain diseases could affect the results of the surgery. Moreover, the comparison between outcomes of minimally invasive decompressive surgeries for stenosis on different levels of the spine is also of important value in assessing the efficacy of this type of surgery and its advantage over other surgeries.

This study offers several perspectives for further investigations. The findings reached in this study are of added value to the current practice, especially in Lebanon, where advancements in surgical techniques are not applied directly. The positive outcomes of minimally invasive bilateral decompression of lumbar stenosis via a unilateral approach under spinal anesthesia shed light on the need for further investigations, locally and globally, to reach better practice with better outcomes.

Long-term follow-up is necessary to better evaluate the results of this type of surgery and to compare it to other surgeries. Longitudinal studies tracking patients over extended periods can provide insights into the durability of outcomes. This can elucidate the long-term effectiveness and potential recurrence rates associated with this technique. In addition, comparative studies are also required to better compare the outcomes of minimally invasive unilateral decompression with those of traditional open surgery or other minimally invasive techniques. Understanding the relative benefits and drawbacks of various surgical options can guide treatment decisions and optimize patient care.

Other points worth investigating are the factors associated with treatment success, such as including patients with specific cases. For instance, the efficiency of this surgical technique in patients having bone or joint disorders is worth studying. Also, identifying predictors of positive outcomes can help tailor treatment approaches to individual patient needs and improve overall surgical success.

Another interesting point to assess is the effect of this technique on health economics. Health economic analyses, targeting the cost-effectiveness of each technique, can inform healthcare resource allocation and reimbursement policies.

On the technological level, research related to surgical techniques, instrumentation, and imaging modalities can further enhance the efficacy and safety of minimally invasive decompression surgery, leading to refinements in surgical approaches, shorter operative times, and improved patient outcomes.

## Conclusions

To our knowledge, this study was the first in Lebanon to assess minimally invasive techniques. It not only contributed valuable insights to the global body of literature but also addressed a critical gap in the local healthcare landscape. Our findings provide important evidence for the efficacy and benefits of this surgical technique within the Lebanese context, offering clinicians and patients a novel treatment option for lumbar stenosis. Furthermore, the successful implementation of this study underscores the capacity and expertise of Lebanese healthcare professionals in adopting and advancing innovative surgical approaches. Based on the impressive results reached following this type of surgery, this study sheds light on the need for further studies that compare the outcomes to different surgery types, explore long-term outcomes, refine patient selection criteria, and enhance technological advancements in surgical practice. Moreover, this study can serve as a foundation for future research and clinical practice in Lebanon, guiding healthcare providers in the adoption of minimally invasive techniques and optimizing patient care pathways. Additionally, our findings may inspire further collaboration and knowledge exchange among researchers, clinicians, and healthcare institutions. 

## Appendices

Appendix 1 

Surgical Technique

Position and incision: As a start, an image intensifier for a precise incision and a retractor to identify the inferior part of the superior lamina are necessary. To increase the surgical side clearance, the patient is positioned laterally on the operating table with the target segment (Figures 1, 2). An X-ray image is used to confirm the level of decompression. For the safety line, the projection of the transverse process of the adjacent vertebral bodies on the body surface is used in the lateral radiograph. The lines between the apex of the upper process of the lower vertebral body and the posterior upper corner of the lower vertebral body are marked, where the intersection of these lines is the point where the needle should enter. 

After disinfecting the area, an anesthetic, such as Lidocaine, is administered to each of the layers. Around a 20mm skin incision is enough to access the two segments targeted for decompression. It is important to dilate each level of the spinal cord to preserve the multifidus muscle inserted along the spinous process between adjacent levels. The skin incision is usually made lateral to the midline for L4-L5-S1, and more medially for the upper levels to conserve the pars interarticularis. Despite the insertion of large-diameter dilators, smaller tubes are used to avoid any violation of the pars interarticularis (Figures 3, 4).

Bone removal, exposure, and decompression: The soft tissue is removed to expose the bony structures and for easier identification of the inferior edge of the lamina. The ligamentum flavum is easily reached through a drill after the removal of the ipsilateral medial and inferior portion of the lamina. After the cranial insertion of the ligamentum flavum is identified, the ipsilateral one is removed. Usually, the superior aspect of the decompression is linked to the superior ligamentum flavum. However, in certain cases, for instance, when the facet joint cysts extend beyond the limits of the ligamentum flavum, the upper limit of the latter is removed at first for a better landmark confirmation of the superior limit of the decompression.

Access to the contralateral side from the ipsilateral approach: For better visualization, the operating table is tilted. Bilateral decompression is initiated by partial undercutting of the base of the spinous process to access the midline and the contralateral portion of the ligamentum flavum, while preserving the tip and dorsal aspect of the spinous process as well as the supraspinous and interspinous ligaments, thereby maintaining the posterior tension band. On the ipsilateral side, the hypertrophied ligamentum is removed and the thecal sac identified. The thecal sac, a membranous sheath surrounding the spinal cord, may be compressed in stenosis and lead to symptoms such as cauda equina syndrome with bladder or rectal dysfunction. Further decompression is achieved by removing the upper portion of the caudal lamina, which facilitates canal expansion and reduces the risk of recurrent stenosis. Decompression of the thecal sac is then performed in a clockwise fashion, beginning at the 6 o’clock position under microscopic guidance. This maneuver carries a higher risk of dural injury or CSF leak; therefore, adequate space on the ipsilateral side is created to safely reach the contralateral aspect. The same steps are repeated on the contralateral side to complete bilateral decompression.

Hemostasis and closure: The MIS is associated with minimal blood loss (10 to 20 mL). However, blood loss control is essential for a better success of the successful operation. Despite that, bone bleeding is rare; bone wax should be used in case of any complication.

Concerning the closure, the insertion of a drain is not common. The closure targets mainly the thoracolumbar fascia. In some cases, the ipsilateral muscle is injected at the end with local anesthetic for a significant reduction of postoperative pain. Fat and skin closure are the final steps of the operation and can be done in different ways, depending on the surgeon.

Appendix 2

**Table 9:** Power calculation details.

Input:	Tail(s)	=	Two
	Effect size	=	0.9153404
	α err prob	=	0.001
	Power (1-β err prob)	=	0.8
Output:	Noncentrality parameter	=	4.6673386
	Critical t	=	1.959964
	Df	=	25
	Total sample size	=	26
	Actual power	=	0.8072813

Appendix 3

**Table 10:** Questions for patient assessment. The table is an adapted version of the Oswestry Disability Index (ODI). Approval for use with declaration of authorship and modifications has been granted to the authors and is available upon request.

Section	Question	Response Options
A. Sociodemographics	Gender	Male; Female
A. Sociodemographics	Age	Free text
A. Sociodemographics	Marital status	Single; Married; Divorced; Widowed
A. Sociodemographics	Residency Location	North; South; Beirut; Bekaa; Mount Lebanon
A. Sociodemographics	Education Level	Not educated; School education; Healthcare-related education; Non-healthcare related education
A. Sociodemographics	Occupation	Unemployed; Physical effort; Little or no physical effort
B. Before Surgery - Quality of Life Assessment	Pain intensity	No pain; Very mild; Moderate; Fairly severe; Very severe; Worst imaginable
B. Before Surgery - Quality of Life Assessment	Personal Care	Normal without pain; Normal with pain; Painful and slow; Some help; Daily help; Cannot care at all
B. Before Surgery - Quality of Life Assessment	Lifting	Heavy w/o pain; Heavy with pain; Only if positioned; Light/medium positioned; Very light; Cannot lift
B. Before Surgery - Quality of Life Assessment	Walking	No limit; >2 km; >1 km; >500 m; With aid; Bedridden
B. Before Surgery - Quality of Life Assessment	Sitting	Any chair; Favorite chair; <1 hour; <30 min; <10 min; Cannot sit
B. Before Surgery - Quality of Life Assessment	Standing	No pain; With pain; <1 hour; <10 min; <3 min; Cannot stand
B. Before Surgery - Quality of Life Assessment	Sleeping	Never disturbed; Occasionally disturbed; <6 hrs; <4 hrs; <2 hrs; No sleep
B. Before Surgery - Quality of Life Assessment	Social Life	Normal; Normal with pain; Energetic limited; Restricted outings; Homebound; None
B. Before Surgery - Quality of Life Assessment	Travelling	Anywhere no pain; Anywhere with pain; >2 hrs; <1 hr; <30 min; Only for treatment
C. After Surgery - Quality of Life Assessment	Pain intensity	No pain; Very mild; Moderate; Fairly severe; Very severe; Worst imaginable
C. After Surgery - Quality of Life Assessment	Personal Care	Normal without pain; Normal with pain; Painful and slow; Some help; Daily help; Cannot care at all
C. After Surgery - Quality of Life Assessment	Lifting	Heavy w/o pain; Heavy with pain; Only if positioned; Light/medium positioned; Very light; Cannot lift
C. After Surgery - Quality of Life Assessment	Walking	No limit; >2 km; >1 km; >500 m; With aid; Bedridden
C. After Surgery - Quality of Life Assessment	Sitting	Any chair; Favorite chair; <1 hour; <30 min; <10 min; Cannot sit
C. After Surgery - Quality of Life Assessment	Standing	No pain; With pain; <1 hour; <10 min; <3 min; Cannot stand
C. After Surgery - Quality of Life Assessment	Sleeping	Never disturbed; Occasionally disturbed; <6 hrs; <4 hrs; <2 hrs; No sleep
C. After Surgery - Quality of Life Assessment	Social Life	Normal; Normal with pain; Energetic limited; Restricted outings; Homebound; None
C. After Surgery - Quality of Life Assessment	Travelling	Anywhere no pain; Anywhere with pain; >2 hrs; <1 hr; <30 min; Only for treatment
D. About the Surgery	Operation time	Free text
D. About the Surgery	Bleeding	Free text
D. About the Surgery	Accidental dural tear	Yes; No
D. About the Surgery	Post-op Infection	Yes; No
D. About the Surgery	If yes, elaborate	Free text
E. Claudication Pain Assessment	Rate claudication pain	0: No pain; 1: Minimal; 2: Moderate; 3: Intense; 4: Maximal
